# Supplementary material for: Phase Behavior of NR/PMMA Semi-IPNs and Development of Porous Structures
Source: Polymers (Basel). 2023 Mar 8;15(6):1353. doi: 10.3390/polym15061353 (PMC10058802; doi:10.3390/polym15061353)
Supplement: Supplementary file 1 [file polymers-15-01353-s001.zip › polymers-2233908-supplementary.pdf]

polymers

MDPI

Supplementary Material: Phase Behavior of NR/PMMA Semi-IPNs and Development of Porous Structures

Jacob John, Damir Klepac, Mia Kurek, Mario Ščetar, Kata Galić, Srećko Valić, Sabu Thomas, Anitha Pius

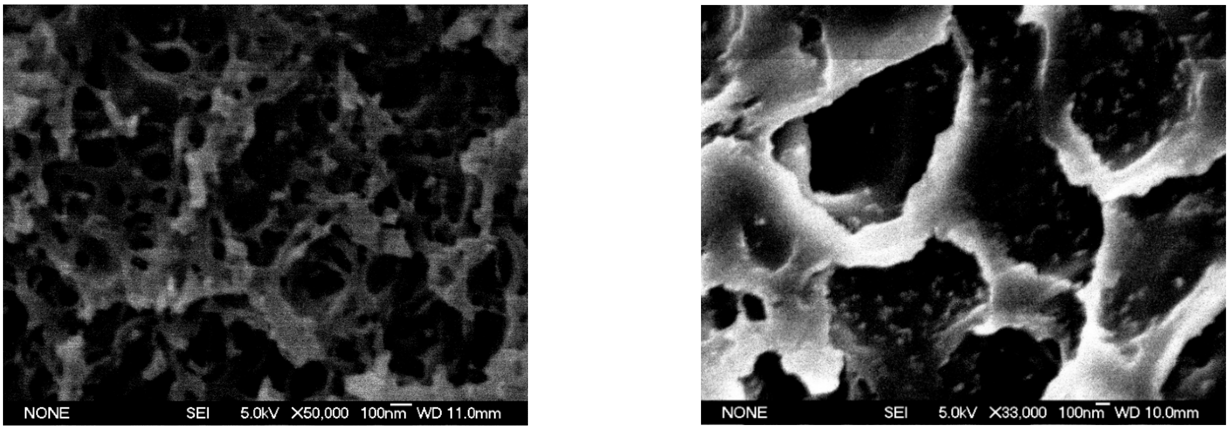

(a) **(b)**

Figure S1. SEM images of blends **(a)** NRM_{50} and **(b)** NRM_{65}.
